# Supplementary material for: Long-Term Immunity against SARS-CoV-2 Wild-Type and Omicron XBB.1.5 in Indonesian Residents after Vaccination and Infection
Source: Antibodies (Basel). 2024 Sep 2;13(3):72. doi: 10.3390/antib13030072 (PMC11417924; doi:10.3390/antib13030072)
Supplement: Supplementary file 1 [file antibodies-13-00072-s001.zip › antibodies-3117874-supplementary.pdf]

## 6. Supplementary Materials

*S1. Comparative Analysis of Serum Antibody Titers between Wild-Type and Omicron XBB.1.5 across Booster and Infection Status Groups.*

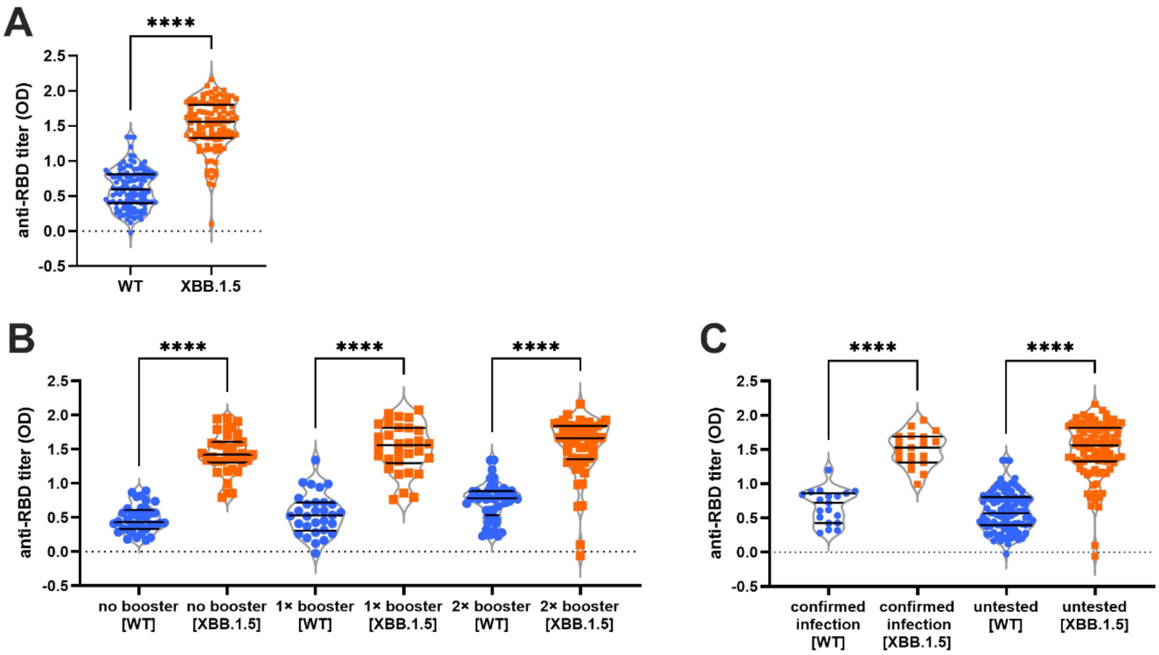


**Supplementary Figure S1.** Significantly higher Omicron XBB.1.5 antibody titers compared to Wild-Type (WT) antibody titers **(A)**. Subgroup comparisons also show consistently higher Omicron XBB.1.5 versus WT antibody titers among people who have not received a booster (no booster), received a single booster (1x booster), and received two boosters (2x booster) for both WT and XBB.1.5, as shown in **(B)**. Anti-RBD XBB.1.5 titer is significantly higher than anti-RBD WT in both pre-infected individuals and untested individuals **(C)**. The antibody titers were measured using indirect ELISA and are expressed as ELISA's optical density (OD) measurements at 414 nm. Individual values are shown, and horizontal lines represent median and quartile of Ab titers. Statistical analysis was performed using the Mann-Whitney U test (A) and the Kruskal-Wallis test (B & C). **** $p<0.0001$, ** $p<0.01$, * $p<0.05$.

*S2. Comparative Serum Neutralization Capacity between Wild-Type and Omicron XBB.1.5 across Booster and Infection Status Groups.*

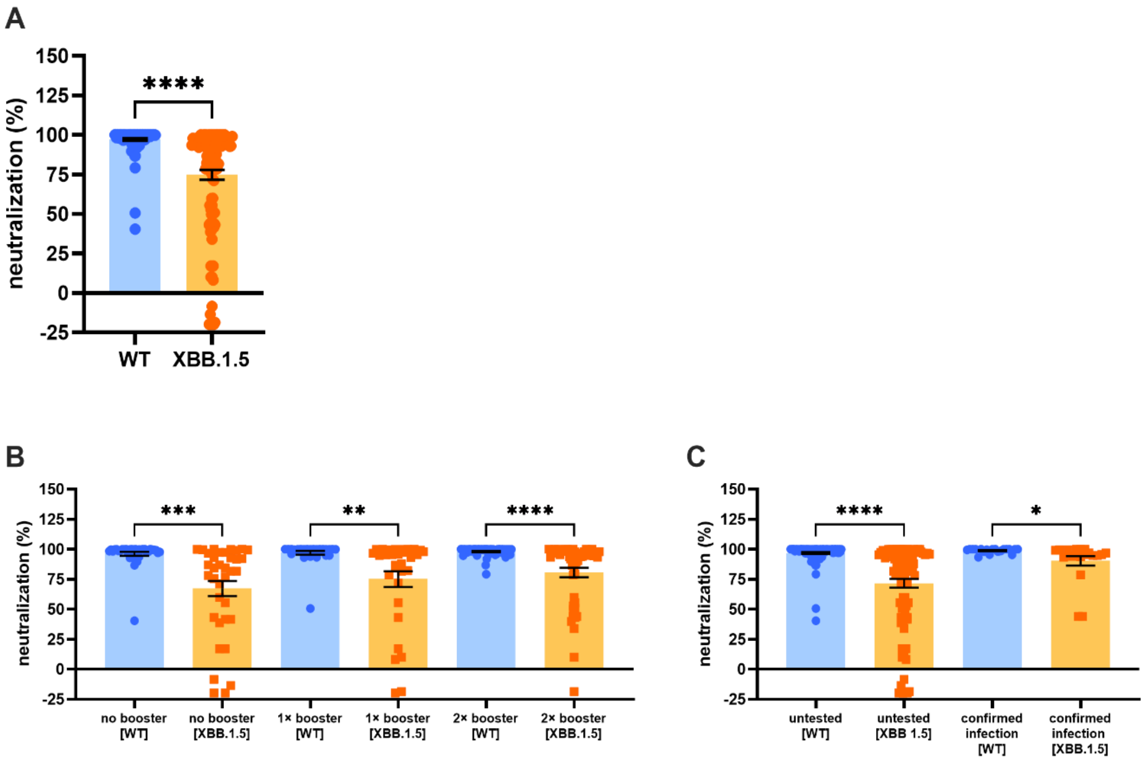


**Supplementary Figure S2.** Significantly higher serum neutralization capacity (NC) of Wild-Type (WT) compared to Omicron XBB.1.5 (A). Similarly, consistent results are also shown in the booster analysis subgroup among people who have not received a booster (no booster), received a single booster (1x booster), and received two boosters (2x booster), for both WT and XBB.1.5 (B). The NC against WT is higher than that against XBB.1.5. in both pre-infected and untested individuals (C). Serum NC was measured using ONE-Glo EX$^{TM}$ Luciferase Assay System. Individual values are shown with bars that represent the means and horizontal lines that represent SEM. Statistical analyses were performed using the Mann–Whitney U test (A) and the Kruskal-Wallis test (B & C). **** $p$<0.0001, ** $p$<0.01, * $p$ < 0.05

*S3. Exploring Correlation Patterns: Serum Antibody Titers and Neutralization Capacity Across Wild-Type and Omicron XBB.1.5 Variants.*

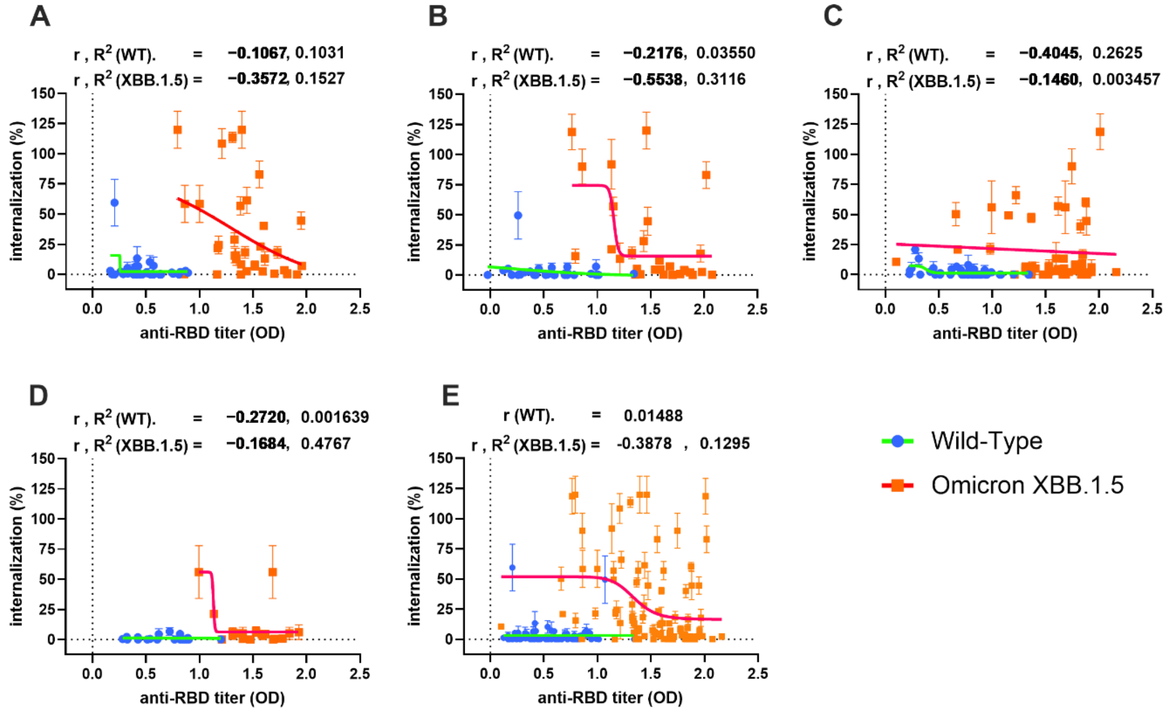


**Supplementary Figure S3.** Subgroup analysis of correlation between serum antibody titers and neutralization capacity (NC) for respective variants. Subgroup analysis by the number of booster doses (A-C) demonstrates a consistent relationship among non-booster recipients (A), single-booster recipients (B), and double-booster recipients (C). Similarly, subgroup analysis by infection status reveals comparable results for individuals with breakthrough infections (D) and untested individuals (E). The antibody titers were measured using indirect ELISA and are expressed as ELISA's optical density (OD) measurements at 414 nm, while the NC was measured using ONE-Glo EX$^{TM}$ Luciferase Assay System and expressed as the % internalization of the pseudovirus. Red lines and green lines depict the non-linear regression model between variables.
